# Supplementary material for: The interactome of intact mitochondria by cross-linking mass spectrometry provides evidence for coexisting respiratory supercomplexes
Source: Mol Cell Proteomics. 2017 Dec 8;17(2):216–32. doi: 10.1074/mcp.RA117.000470 (PMC5795388; doi:10.1074/mcp.RA117.000470)
Supplement: Supplemental Data [file supp_RA117.000470_133922_0_supp_23978_fzffwf.zip › spectra_annotation/mito_DR_spectra_annotation/146-1-3-1-9-1.pdf]

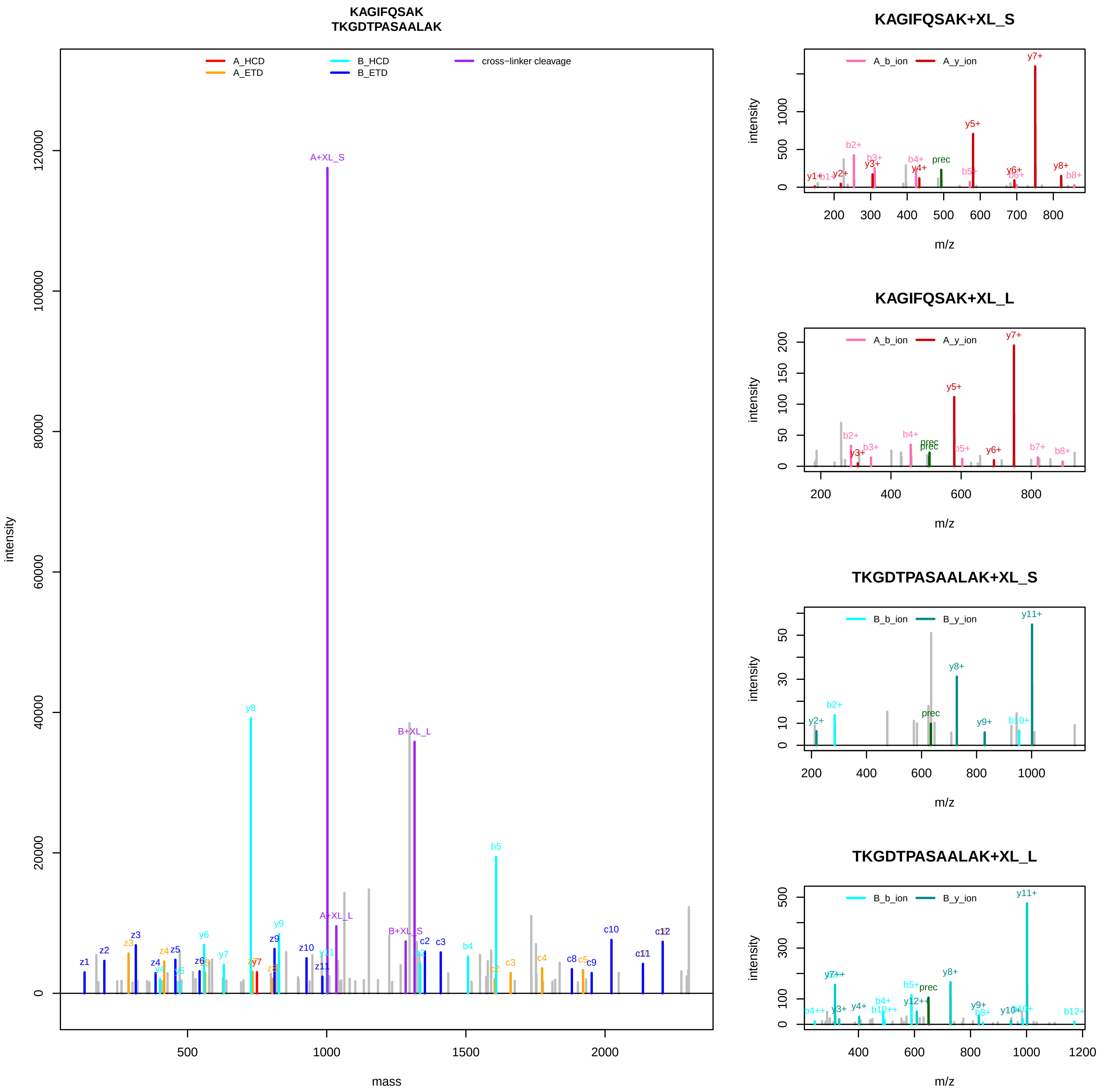
KAGIFQSAK
TKGDTPASAALAK
A_HCD
A_ETD
B_HCD
B_ETD
cross−linker cleavage
A+XL_S
B+XL_L
A+XL_L
B+XL_S
y8
b5
y9
y6
y7
z1
z2
z3
z4
z5
z6
z9
z10
z11
y11
c2
c3
c4
c5
c8
c9
c10
c11
c12
b4
intensity
mass
KAGIFQSAK+XL_S
A_b_ion
A_y_ion
y7+
y5+
b2+
prec
y8+
m/z
KAGIFQSAK+XL_L
TKGDTPASAALAK+XL_S
B_b_ion
B_y_ion
y11+
y8+
b2+
y2+
y9+
b10+
TKGDTPASAALAK+XL_L
y12++
b5+
b12+
